# The Significance of the Epiphyses

**Published:** 1910-09-17

**Authors:** 


### The Significance of the Epiphyses.

The well-known American authority on children's diseases, Dr. T. Morgan Rotch, of Boston, has read a paper before the annual meeting of the American Pediatric Society * in session at Washington, which is especially interesting when considered and compared wth the forecasts of Professor Geddes, briefly discussed in this journal under the title of "The Aims of Anatomy" (The Hospital, January 1, 1910, p. 377). Those who were interested in the ingenious previsions of the Dublin anatomist will recall that the feature of his introductory lecture at the Royal College of Surgeons in Ireland was the importance which he attaches to the correlation of structure and function in the human frame. One of the examples which he gave of the curious and little-understood relations of apparently disconnected organs of the body was the fact that it is possible from the proportional lengths of some of the limb bones of adults to judge the activity of the reproductive glands. Whether Dr. Rotch has been influenced by the ideas which Professor Geddes has propounded we do not know; but certainly he is making a beginning along those lines which the latter regards as marking the future progress and aims of anatomy.

Dr. Rotch presents so far only a preliminary outline of the conclusions to which his researches are leading him. Professor Pryor, another American investigator, is also working in the same field, and his results support those of the Boston pediatrist. The latter bases his conclusions upon some hundreds of *x*-ray observations which he has carried out upon children, with especial reference to the epiphyses; and he believes that the development of these structures can serve as a means for classifying individuals from birth to the completion of development. Individuals fall into groups not chronologically but by a graded set of developmental epiphyses. When boys and girls are thus graded it is seen that the groups have little to do with either age, height, or weight. The charts of epiphyseal development enunciate strongly the lack of correlation between epiphyseal growth and general physical development. Moreover, he says, epiphyseal growth has a certain bearing on mental growth, which leads on to the idea that an epiphyseal standard should be substituted for chronological ones in dealing with children. The child's general welfare depends to a certain degree on keeping its mental and epiphyseal development in equilibrium, thus avoiding overstrain, both mental and physical. In this hypothesis, astonishing as it looks at first sight, there is really much that is reasonable. Nothing is more certain than that age, height, and weight as tests of the degree of mental development of a child or adolescent are almost valueless. If, therefore, it can be shown that in epiphyseal development a more certain guide exists, there is no doubt that its adoption would be very useful. The conception is ingenious, and the tentative manner in which it has been put forward shows that its author realises that there is a great deal of work to be done before it can meet with general acceptance. But as an earnest of commencing progress in the science of anatomy towards the ideal which Professor Geddes last year sketched out, this suggested epiphyseal or anatomical standard for the education of children is stimulating and worthy of emulation.

It is only to be expected that the application of this principle, should it turn out after further investigation to be trustworthy, will be ridiculed and opposed if it is pushed too hastily to its logical conclusion. The idea of judging a candidate's fitness for admission to the Civil Service, let us say, or the Army, or any of the learned professions, rather by the *x*-ray demonstration of the state of his bones than by his actual age is one which the lay Press is only too likely to regard as an occasion for hilarity. And indeed the English mind is too essentially conservative for even medical men to espouse Dr. Rotch's theories with any degree of enthusiasm at first. Much remains to be done before anything in the nature of scientific proof can be offered as to their correctness or otherwise; but the "wildest dreams of Kew are the facts of Khatmandu," and the vainest dreams of one generation are so often the commonplaces of the next that it does not do to scout these ingenious ideas merely because of their unfamiliarity.

* "Archives of Pediatrics," August, 1910, p. 567.

